# Cardiac magnetic resonance–derived myocardial scar is associated with echocardiographic response and clinical prognosis of left bundle branch area pacing for cardiac resynchronization therapy

**DOI:** 10.1093/europace/euad326

**Published:** 2023-10-31

**Authors:** Zhongli Chen, Xuan Ma, Yuan Gao, Sijin Wu, Nan Xu, Feng Chen, Yanyan Song, Chongqiang Li, Minjie Lu, Yan Dai, Michael R Gold, Shihua Zhao, Keping Chen

**Affiliations:** State Key Laboratory of Cardiovascular Disease, Cardiac Arrhythmia Center, Fuwai Hospital, National Center for Cardiovascular Disease, Chinese Academy of Medical Sciences and Peking Union Medical College, No. 167 North Lishi Rd, Xicheng District, Beijing 100037, China; Department of Magnetic Resonance Imaging, National Center for Cardiovascular Diseases, Fuwai Hospital, Chinese Academy of Medical Sciences and Peking Union Medical College, No. 167 North Lishi Rd, Xicheng District, Beijing 100037, China; State Key Laboratory of Cardiovascular Disease, Cardiac Arrhythmia Center, Fuwai Hospital, National Center for Cardiovascular Disease, Chinese Academy of Medical Sciences and Peking Union Medical College, No. 167 North Lishi Rd, Xicheng District, Beijing 100037, China; State Key Laboratory of Cardiovascular Disease, Cardiac Arrhythmia Center, Fuwai Hospital, National Center for Cardiovascular Disease, Chinese Academy of Medical Sciences and Peking Union Medical College, No. 167 North Lishi Rd, Xicheng District, Beijing 100037, China; Department of Echocardiography, National Center for Cardiovascular Diseases, Fuwai Hospital, Chinese Academy of Medical Sciences and Peking Union Medical College, No. 167 North Lishi Rd, Xicheng District, Beijing 10037, China; State Key Laboratory of Cardiovascular Disease, Cardiac Arrhythmia Center, Fuwai Hospital, National Center for Cardiovascular Disease, Chinese Academy of Medical Sciences and Peking Union Medical College, No. 167 North Lishi Rd, Xicheng District, Beijing 100037, China; Department of Magnetic Resonance Imaging, National Center for Cardiovascular Diseases, Fuwai Hospital, Chinese Academy of Medical Sciences and Peking Union Medical College, No. 167 North Lishi Rd, Xicheng District, Beijing 100037, China; Catheterization Laboratory, National Center for Cardiovascular Diseases, Fuwai Hospotal, Chinese Academy of Medical Sciences and Peking Union Medical College, No. 167 North Lishi Rd, Xicheng District, Beijing 10037, China; Department of Magnetic Resonance Imaging, National Center for Cardiovascular Diseases, Fuwai Hospital, Chinese Academy of Medical Sciences and Peking Union Medical College, No. 167 North Lishi Rd, Xicheng District, Beijing 100037, China; State Key Laboratory of Cardiovascular Disease, Cardiac Arrhythmia Center, Fuwai Hospital, National Center for Cardiovascular Disease, Chinese Academy of Medical Sciences and Peking Union Medical College, No. 167 North Lishi Rd, Xicheng District, Beijing 100037, China; Division of Cardiology, Medical University of South Carolina, Charleston, SC, USA; Department of Magnetic Resonance Imaging, National Center for Cardiovascular Diseases, Fuwai Hospital, Chinese Academy of Medical Sciences and Peking Union Medical College, No. 167 North Lishi Rd, Xicheng District, Beijing 100037, China; State Key Laboratory of Cardiovascular Disease, Cardiac Arrhythmia Center, Fuwai Hospital, National Center for Cardiovascular Disease, Chinese Academy of Medical Sciences and Peking Union Medical College, No. 167 North Lishi Rd, Xicheng District, Beijing 100037, China

**Keywords:** LBBAP, CMR, Scar, Cardiac resynchronization, Response

## Abstract

**Aims:**

Left bundle branch area pacing (LBBAP) is a novel approach for cardiac resynchronization therapy (CRT), but the impact of myocardial substrate on its effect is poorly understood. This study aims to assess the association of cardiac magnetic resonance (CMR)–derived scar burden and the response of CRT via LBBAP.

**Methods and results:**

Consecutive patients with CRT indications who underwent CMR examination and successful LBBAP-CRT were retrospectively analysed. Cardiac magnetic resonance late gadolinium enhancement was used for scar assessment. Echocardiographic reverse remodelling and composite outcomes (defined as all-cause death or heart failure hospitalization) were evaluated. The echocardiographic response was defined as a ≥15% reduction of left ventricular end-systolic volume. Among the 54 patients included, LBBAP-CRT resulted in a 74.1% response rate. The non-responders had higher global, septal, and lateral scar burden (all *P* < 0.001). Global, septal, and lateral scar percentage all predicted echocardiographic response [area under the curve (AUC): 0.857, 0.864, and 0.822; positive likelihood ratio (+LR): 9.859, 5.594, and 3.059; and negative likelihood ratio (−LR): 0.323, 0.233, and 0.175 respectively], which was superior to QRS morphology criteria (Strauss left bundle branch abnormality: AUC: 0.696, +LR 2.101, and −LR 0.389). After a median follow-up time of 20.3 (11.5–38.7) months, higher global, lateral and septal scar burdens were all predictive of the composite outcome (hazard ratios: 4.996, 7.019, and 4.741, respectively; *P*’s < 0.05).

**Conclusion:**

Lower scar burden was associated with higher response rate of LBBAP-CRT. The pre-procedure CMR scar evaluation provides further useful information to identify potential responders and clinical outcomes.

## Graphical Abstract

**Graphical Abstract:**
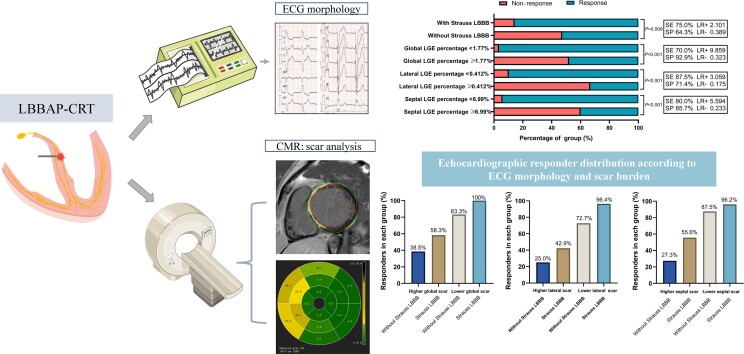
Cardiac magnetic resonance–derived myocardial scar predicts response of cardiac resynchronization therapy via left bundle branch area pacing. Baseline electrocardiogram morphology and pre-procedural scar evaluation from cardiac magnetic resonance predict reverse remodelling after left bundle branch area pacing for cardiac resynchronization therapy. Low global, septal, and lateral scar burden helps to identify more responders on top of Strauss left bundle branch abnormality.

**What’s new?**

- Global, septal, and lateral scar burdens in the left ventricle correlated negatively with reverse remodelling and displayed better performance beyond left bundle branch abnormality for predicting echocardiographic response in patients who underwent left bundle branch area pacing for cardiac resynchronization therapy (LBBAP-CRT).
- Measures of myocardial scar were also strong predictors of clinical outcomes after LBBAP-CRT.
- Pre-procedure cardiovascular magnetic resonance for myocardial scar evaluation may offer values to improve the selection of LBBAP-CRT candidates and optimize clinical decision-making.

## Introduction

Cardiac resynchronization therapy (CRT) with traditional biventricular pacing (BVP) is an established effective therapy for patients with heart failure (HF) with a reduced ejection fraction (EF) and QRS prolongation, despite optimal medical therapy. There is a 30–40% non-responding rate using traditional criteria,^1,2^ although the classification of response remains controverisal.^3,4^ More recently, techniques have been developed to perform left bundle branch area pacing (LBBAP), as a rescue or alternative for BVP.^5,6^ Through recruitment of the intrinsic left ventricular (LV) conduction system, LBBAP can also achieve improved electrical and mechanical synchrony, especially in patients with left bundle branch abnormality (LBBB).^7^ Multicentre studies have reported both acute and long-term safety and efficacy of this approach.^5,8^ More recently, a prospective, randomized trial (LBBP-RESYNC) with a head-to-head comparison of LBBAP-CRT and BVP-CRT showed LBBAP as a promising alternative to traditional CRT. There was greater LVEF improvement compared with BVP-CRT among patients with LBBB and non-ischaemic HF.^9^

In contrast to BVP-CRT, there are still no tailored criteria for patient selection, and established evidence for response rate is limited. Based on standard BVP-CRT selection criteria, such as LV systolic dysfunction, prolonged QRS duration (QRSd), and LBBB morphology, the non-responder rate for LBBAP-CRT ranges from 10 to 30%.^5,10,11^ Apart from these clinical parameters noted above, pre-procedure imaging evaluation of LV was considered an important quality indicator to improve the care and outcomes of cardiac pacing^12^; however, limited data exist regarding the imaging evaluation in relation to the LBBAP-CRT response and HF prognosis.

Cardiovascular magnetic resonance (CMR) by late gadolinium enhancement (LGE) is a well-established and accurate approach for localizing and quantifying myocardial scar. In BVP-CRT, scar in the LV pacing site has been associated with adverse outcomes following implantation.^13^ However, the effect of the scar on LBBAP-CRT acute response and prognosis is unknown. Moreover, since LBBAP is commonly achieved through deep septal penetration, so there is concern that septal scar may be detrimental on the response of LBBAP-CRT. A recent study indicates that CMR helps in predicting the procedural failure of LBBAP among patients with extensive LV scar burden.^14^ Data from computer simulations predict that septal scar may also have an adverse impact on LBBAP-CRT response.^15^ However, there is a paucity of clinical data concerning the prognostic impact of CMR-LGE for LBBAP among HF patients with CRT indications. Accordingly, in the present study, the impact of scar features on LBBAP-CRT response was evaluated including acute and more long-term clinical outcomes.

## Methods

### Study population

This was a retrospective, single-centre study. Consecutive patients were enrolled from January 2019 to March 2022, who had New York Heart Association (NYHA) functional classes II–IV, HF symptoms despite optimal guideline-directed medical therapy, baseline LVEF ≤35% with indications for CRT or baseline LVEF <50% with expected high ventricular pacing burden >40%,^16,17^ and underwent CMR examination (cine and contrast) prior to the CRT implantation within 3 months of implantation. Exclusion criteria were listed as follows: (i) age < 18 years, (ii) life expectancy <12 months, (iii) pre-existing CRT devices or pacemakers, (iv) without available pre-procedural CMR examination in our centre, and (v) CMR image quality not allowing accurate LGE analysis. A flow chart of patients meeting exclusion criteria is presented in Supplementary material online, *Figure S1*.

In our centre, for patient indicated for CRT who had LBBB morphology or with expected high ventricular pacing percentage, LBBAP was mainly taken as a primary strategy. For the CRT candidates with prolonged QRSd but did not meet the LBBB criteria, BVP was often preferred as the primary strategy, with LBBAP serving as a rescue approach. Patients with successful LBBAP as a primary CRT approach (*n* = 43) or rescue approach (*n* = 11) to failed BVP because of difficult coronary sinus (CS) lead placement or high capture threshold comprised the study population. The pre-procedure standard clinical information including NYHA functional class, comorbidities, medication therapy, serum blood test results, and echocardiographic assessment were collected. In this study, LBBB was defined according to the Strauss criteria, as QRSd >140 ms in men (>130 ms in women) and the presence of at least two mid-QRS notches or slurs in leads I, aVL, V1, V2, V5, and V6.^18^ The study conforms to the Helsinki Declaration guidelines and was approved by the Fuwai Hospital Institutional Review Board and Ethical Committee (Approval No. 2019-1149). All patients provided informed consent for the procedure.

### Procedure details and device programming

Left bundle branch area pacing implantation was performed as previously described.^10^ In brief, the SelectSecure pacing lead (Model 3830 69 cm, Medtronic Inc., Minneapolis, MN, USA) was introduced in the right ventricle through the fixed-curve sheath delivery system (C315 HIS, Medtronic Inc., Minneapolis, MN, USA) under the fluoroscopic right anterior oblique 30°. The lead was advanced into the ventricular septum from the right side of interventricular septum towards the LBB area. Real-time 12-lead electrocardiograms (ECG) and intracardiac electrograms were recorded during the procedure and pacing tests. QRS morphology was monitored, and the pacing stimulus to R wave peak time (stim-RWPT) in lead V6 was measured at both low and high output. Left bundle branch capture was considered present according to previous definitions.^5,19,20^

For those patients implanted with a CRT-pacemaker (CRT-P) device (*n* = 16), the 3830 lead was connected to the right ventricular (RV) port, with the CS lead connecting to the LV port as a backup. For those implanted with a CRT-defibrillator (CRT-D) device, the 3830 lead was connected to the LV port, and the defibrillator lead was connected to the RV port. The atrioventricular (AV) and/or interventricular (VV) delay was optimized to ensure the narrowest QRS complex in LBBAP-CRT. If the electrical correction was favourable by LBBAP, the CS-LV lead was inactivated allowing for LBBAP alone. Four patients received LBBAP and LV pacing (LOT-CRT) due to unfavourable electrical resynchronization.

### Cardiovascular magnetic resonance acquisition

Standard CMR was performed on 3.0-T scanners (Discovery MR750W, GE Healthcare, Milwaukee, WI; Ingenia, Philips Healthcare, Best, The Netherlands; or Skyra, Siemens, Erlangen, Germany) with a phased-array cardiac coil and retrospective electrocardiographic gating. Cine images using balanced steady-state free precession (bSSFP) were acquired in three long-axis planes (LV outflow tract, two- and four-chamber view) and in sequential short-axis slices from the atrioventricular ring to the LV apex.

### Cardiovascular magnetic resonance analysis

All CMR images were uploaded and analysed using commercial post-processing software CVI42 (version 5.12.4, Circle Cardiovascular Imaging, Calgary, Canada). The images were reviewed separately by two radiologists (Y.S. and X.M.) who were blinded to the clinical information or outcomes and adjudicated by the senior radiologist (S.Z.) to minimize the difference.

For LV deformation analysis, end-diastolic endo- and epicardial contours were traced semi-automatically with manual adjustment in long-axis views and short-axis views on cine images.^21^ Late gadolinium enhancement images were first reviewed for visible LGE (areas with relatively increased signal intensity following administration of gadolinium contrast), and if positive, its location and pattern were categorized. The location was classified as septal, LV free wall, or as occurring in both locations. The pattern was classified as linear mid-wall, subepicardial, focal, or as occurring in multiple patterns. Late gadolinium enhancement quantification was performed by setting the signal intensity threshold at 6 SD above the mean intensity of a reference region of myocardium that had no visual evidence of enhancement. The manual correction was performed for obvious threshold errors. The percentage of LGE is presented as the percentage of total LV mass (LGE%). In addition to global quantification of LGE at this level, segmental quantification was performed based on the American Heart Association 16-segment model after defining the reference point at RV insertion. The percentage of LGE in anteroseptal and anteroseptal (2, 3, 8, and 9) segments was used to calculate ‘septal LGE%’, while anterolateral and inferolateral (5, 6, 11, and 12) segments were used to calculate ‘lateral LGE%’.

Further details of the LBBAP-CRT implantation and the CMR acquisition are presented in the Supplementary material.

### Outcome definition and follow-up

All patients received routine clinic follow-ups at 6 months for NYHA functional class and echocardiographic indices. The primary echocardiographic response was defined as ≥15% LV end-systolic volume (LVESV) reduction in accordance with previous publications.^22,23^ Super-response was defined as an absolute improvement of LVEF ≥ 20% or LVEF to ≥50% for patients with baseline LVEF ≤35%.^5^ In addition, the percentage change in LVESV, LVEF, and LV end-diastolic diameter (LVEDD) were measured at baseline and 6 months. Clinical response at 6 months was defined as an improvement in NYHA functional class by at least one class and no HF hospitalization (HFH).^24^ Patients were followed up for the composite outcome of any HFH or all-cause death by trained doctors on a regular phone interview or outpatient service. Heart failure hospitalization was defined as a hospital admission or an urgent care visit for intensive treatment for HF with intravenous diuretics or intravenous inotropic medications. The last follow-up time was in November 2022.

### Statistical analysis

Continuous variables were described as mean with standard deviation if normally distributed, otherwise as median with interquartile range (IQR). The discrete variables were depicted as counts and proportions. Student’s *t*-test/Mann–Whitney *U* test was applied for evaluating the differences of continuous variables for appropriateness. The *χ*^2^ test or Fisher’s exact test was used for assessing the difference of discrete variables between groups. The correlation between imaging markers and the change of LVEDD, LVESV, and LVEF was examined by the Spearman correlation rank test and evaluated by the correlation coefficient and *P*-value. A stepwise multivariate linear regression model was used to estimate the contributions of clinical and CMR variables to LVEF improvement. The parameters included in the analysis were age, sex, baseline QRSd, baseline LVEDD, Strauss LBBB, QRSd reduction, stim-V6 RWPT at 3 V at 0.5 ms, the capture of LBB, comorbid atrial fibrillation, and estimated glomerular filtration rate (eGFR),^5,25^ as well as CMR parameters.

The receiver operating characteristic (ROC) curves were performed to evaluate the discriminability of clinical and imaging markers for predicting echocardiographic response, clinical response, and super-response by using the highest Youden index (sensitivity + specificity − 1) to determine the optimal cut-off values for each imaging marker. The positive predictive value (PPV), negative predictive value (NPV), positive likelihood ratio (+LR), and negative likelihood ratio (−LR) were calculated. Kaplan–Meier curves were used to examine cumulative event rates following LBBAP-CRT, and the difference between groups was tested using a log-rank test. The hazard ratio (HR) was estimated using the univariate Cox proportional hazards model. All tests were two tailed with an *α* level of 0.05 considered statistically significant. Statistical analyses were performed using R software version 4.1.2 and SPSS version 22 (IBM, Armonk, NY, USA).

## Results

### Patient characteristics

Fifty-four patients (mean age of 57.7 years old, 53.7% male) were included in this study, including 9 (16.7%) HF patients with expected high ventricular pacing burden due to AV block. Baseline characteristics and echocardiographic evaluation during follow-up of the cohort are summarized in *Table 1* and Supplementary material online, *Table S1*, respectively. Most patients had non-ischaemic cardiomyopathy (NICM, 96.3%). Left bundle branch abnormality morphology meeting Strauss criteria was present in 35 (64.8%) patients, while the remaining 35.2% were composed of intraventricular conduction delay (IVCD; *n* = 14) and narrow QRS complex (*n* = 5). At 6 months follow-up, 40 (74.1%) and 35 (64.8%) patients have echocardiographic and clinical response, respectively, and there were 21 patients (38.9%) classified as super-responders. There was no significant difference in demographic features, pre-procedure LVEF, comorbidities, baseline QRSd, or medical treatment between the responders and non-responders. The patients in the non-response group were characterized by higher baseline N-terminal pro-B-type natriuretic peptide (NT-proBNP) levels, larger LV, advanced NYHA functional class, and without Strauss LBBB ECG morphology. Those with echocardiographic response were more likely to have evidence of LBB capture and a greater QRSd reduction (see Supplementary material online, *Table S1*).

**Table 1:** Clinical characteristics

	All (*n* = 54)	Non-responders (*n* = 14)	Responders (*n* = 40)	*P*-value
Age, years	57.7 ± 11.5	55.4 ± 10.7	58.5 ± 11.8	0.392
Male, *n* (%)	29 (53.7%)	9 (64.3%)	20 (50%)	0.356
Comorbidities				
AF, *n* (%)	8 (14.8%)	4 (28.6%)	4 (10.0%)	0.092
Hypertension, *n* (%)	17 (31.5%)	3 (21.4%)	14 (35.0%)	0.347
CKD, *n* (%)	5 (9.3%)	2 (14.3%)	3 (7.5%)	0.595
ECG parameters				
Baseline QRS duration	166.8 ± 27.0	155.8 ± 35.8	170.6 ± 22.4	0.076
QRS complex morphology				0.027
LBBB (Strauss criteria)	35 (64.8%)	5 (35.7%)	30 (75.0%)	
IVCD	14 (25.9%)	7 (50.0%)	7 (17.5%)	
Narrow QRSd	5 (9.3%)	2 (14.3%)	3 (7.5%)	
Laboratory tests				
eGFR, mL/min	78.4 (68.8–92.1)	70.2 (51.1–85.7)	80.2 (69.4–92.8)	0.15
NT-proBNP, pg/mL	1350 (600.8–2719.1)	2699.2 (1375.2–3747.6)	1181.0 (544.5–2037.8)	0.013
LVEF, %	30 (26.2–35.0)	28.5 (27.0–32.0)	30.5 (26.0–35.0)	0.699
NYHA functional class				
II	13 (24.1%)	1 (7.1%)	12 (30.0%)	0.031
III	39 (72.2%)	11 (78.6%)	28 (70.0%)	
IV	2 (3.7%)	2 (14.3%)	0 (0.0%)	
Medical treatment				
ACE-I/ARB/ARNI, *n* (%)	51 (94.4%)	14 (100.0%)	37 (92.5%)	0.56
Beta blockers, *n* (%)	54 (100.0%)	14 (100%)	40 (100%)	–
Aldosterone antagonists, *n* (%)	53 (98.1%)	14 (100.0%)	39 (97.5%)	1
Diuretics, *n* (%)	53 (98.1%)	14 (100.0%)	39 (97.5%)	1
Digoxin, *n* (%)	21 (38.9%)	6 (42.9%)	15 (37.5%)	0.723
Type of device				0.435
CRT-D, *n* (%)	38 (70.4%)	3 (21.4%)	13 (32.5%)	
CRT-P, *n* (%)	16 (29.6%)	11 (78.6%)	27 (67.5%)	

ACE-I, angiotensin-converting enzyme inhibitor; AF, atrial fibrillation; ARB, angiotensin receptor blockers; ARNI, angiotensin receptor/neprilysin inhibitor; CKD, chronic kidney disease; CRT-D, cardiac resynchronization therapy-defibrillator; CRT-P, cardiac resynchronization therapy-pacemaker; eGFR, estimated glomerular filtration rate; LBBB, left bundle branch abnormality; LVEF, left ventricular ejection fraction; NT-proBNP, N-terminal pro-B-type natriuretic peptide; NYHA, New York Heart Association.

### Cardiovascular magnetic resonance–derived scar features

**Figure 1:**
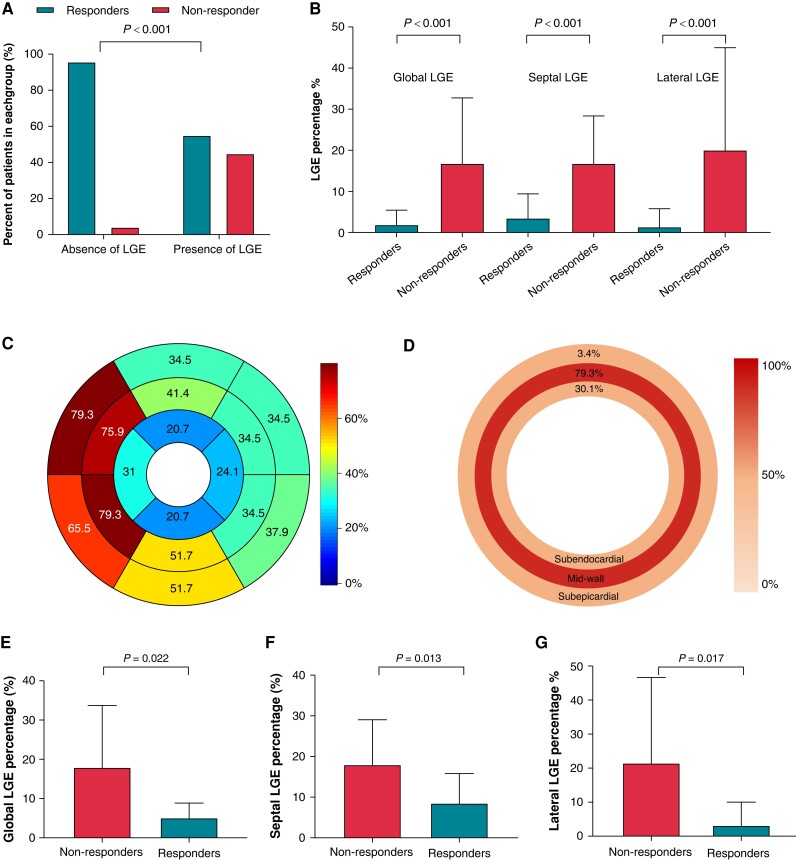
Cardiovascular magnetic resonance–derived scar features and comparison according to echocardiographic response. (*A*) Presence of myocardial scar in relation to echocardiographic response. (*B*) Global, septal, and lateral scar extent according to echocardiographic response in all patients. Barplot depicts mean with standard deviation. (*C*) Distribution of scar among patients with presence of LGE according to the AHA 17 segment. (*D*) Distribution of scar in subendocardial (inner circle), mid-wall (middle circle), and subepicardial layers (outer circle). (*E–G*) Comparison of global, septal, and lateral scar percentage between responders and non-responders in patients with LGE presence. Barplot depicts mean with standard deviation. LGE, late gadolinium enhancement.

Cardiovascular magnetic resonance parameters for the total study population, as well as echocardiographic responder, and non-responder subgroups are summarized in Supplementary material online, *Table S2*. The basic CMR morphological parameters did not demonstrate a significant difference between the responders and non-responders. However, those without myocardial LGE had a higher echocardiographic response rate than those with the presence of LGE (response rate: 96.0% vs. 55.2%, *P* < 0.001; *Figure 1A*). The median percentage of LGE between the responders and non-responders was significantly different, with responders demonstrating lower global, septal, and lateral LGE percentage, as shown in *Figure 1B* and summarized in Supplementary material online, *Table S2*.

In 29 patients with myocardial scar, LGE localized in either free wall (*n* = 4), septal (*n* = 13), or both (*n* = 12), and it was more commonly localized in septal segments (*n* = 25, 86.2%; *Figure 1C*). Patients with combined septal and free-wall LGE displayed were more likely to be non-responders compared with those having LGE located in only one area (*P* = 0.006). Mid-wall LGE was the most common pattern found in the study population, which was observed in 23 patients (79.3%; *Figure 1D*). Among the patients with myocardial scars, global scar percentage [median (IQR) responders: 5.1% (2.0–7.0%) vs. non-responders: 16.6% (4.3–27.0%), *P* = 0.022], lateral scar percentage [median (IQR): responders 0.1% (0.0–1.8%) vs. non-responders: 8.6% (0.7–39.7%), *P* = 0.017] and septal scar percentage [median (IQR): responders 7.4% (3.8–11.8%) vs. non-responders: 17.3% (8.8–22.2%), *P* = 0.013] were also significantly lower in responders than the non-responders (*Figure 1E–G*). The non-responders held significantly higher scar percentage in basal septal segments (*P* = 0.028) and higher scar percentage with a trend toward significance in mid-septal segments (*P* = 0.059).

### Association between scar burden, electrocardiogram morphology, and reverse remodelling

**Figure 2:**
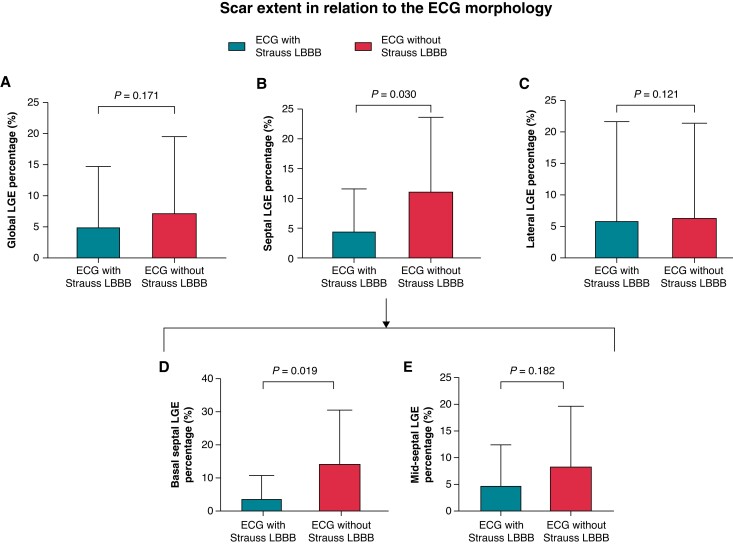
Comparison of scar percentage according to Strauss LBBB. Comparison of global (*A*), septal (*B*), lateral (*C*), basal septal (*D*), and mid-septal (E) LGE percentage between patients with and without Strauss LBBB morphology. Barplots depict mean with standard deviation. LBBB, left bundle branch abnormality; LGE, late gadolinium enhancement.

Septal scar percentage, rather than global or lateral scar percentage, was significantly lower in patients with Strauss LBBB morphology (median 0%, IQR: 0–6.1%) compared with those without Strauss LBBB (median 8.8%, IQR: 0–17.0%, *P* = 0.030). This was especially pronounced in basal septal segments, with rather lower basal septal scar burden in the Strauss LBBB group [median IQR: 0% (0–2.9%) vs. 11.4% (0–20.8%), *P* = 0.019; *Figure 2*]. In correlation analysis, myocardial scar burden correlated negatively with LVEF improvement, LVESV reduction, and LVEDD reduction (all *P* < 0.05; *Table 2*).

**Table 2:** Correlation analysis of CMR scar parameters and reverse remodelling

CMR variables	LVEF improvement		LVESV reduction		LVEDD reduction	
	Correlation coefficients	*P*-value	Correlation coefficients	*P*-value	Correlation coefficients	*P*-value
Global scar percentage, %	−0.519	<0.001	−0.572	<0.001	−0.364	0.007
Lateral scar percentage, %	−0.455	<0.001	−0.577	<0.001	−0.402	0.003
Septal scar percentage, %	−0.543	<0.001	0.580	<0.001	−0.345	0.011

Correlation coefficients were derived from Spearman’s correlation analysis.

LVEDD, left ventricular end-diastolic diameter; LVEF, left ventricular ejection fraction; LVESV, left ventricular end-systolic volume (reduction calculated as percentage of change compared with baseline).

Among the clinical variables, female sex, QRSd reduction, the capture of LBB, baseline LVEDD, and absence of history of atrial fibrillation were all associated with LVEF improvement, while higher baseline LVEDD displayed a negative association with a non-significant trend. The clinical parameters, coupled with CMR parameters, were included in a multivariate linear model with stepwise regression. As a result, QRSd reduction, baseline LVEDD, and septal LGE percentage were independent predictors in the final model of reverse remodelling (*Table 3*). Patients with significant QRSd reduction, smaller LVEDD, and lower septal LGE burden were more likely to have higher LVEF improvement.

**Table 3:** Univariate and multivariate linear regression analyses of baseline determinates of the left ventricular ejection fraction reduction

Variables	Univariate analysis			Multivariate analysis			
	Beta	SE	*P*-value	Beta	SE	VIF	*P*-value
Constant				38.61	11.02		<0.001
Age, years	0.232	0.149	0.126				
Male	−7.557	3.336	0.028				
Baseline QRSd, ms	0.100	0.064	0.131				
Strauss LBBB	4.519	3.597	0.215				
QRSd reduction, ms	0.213	0.095	0.030	0.174	0.086	1.089	0.048
Capture of LBB	6.937	3.354	0.044				
Stim-V6 RWPT	−0.227	0.132	0.092				
Baseline LVEDD, mm	−0.488	0.181	0.009	−0.409	0.166	1.085	0.017
eGFR, mL/min	−0.061	0.084	0.468				
Atrial fibrillation	−10.250	4.698	0.034				
Septal LGE percentage, (%)	−0.624	0.158	<0.001	−0.463	0.159	1.138	0.005
Global LGE percentage, (%)	−0.473	0.152	0.003				
Lateral LGE percentage, (%)	−0.288	0.108	0.010				

LGE, late gadolinium enhancement; SE, standard error; VIF, variance inflation factors; other abbreviations as in *Tables 1* and *2*.

### Prediction of response by scar burden and clinical measures

**Figure 3:**
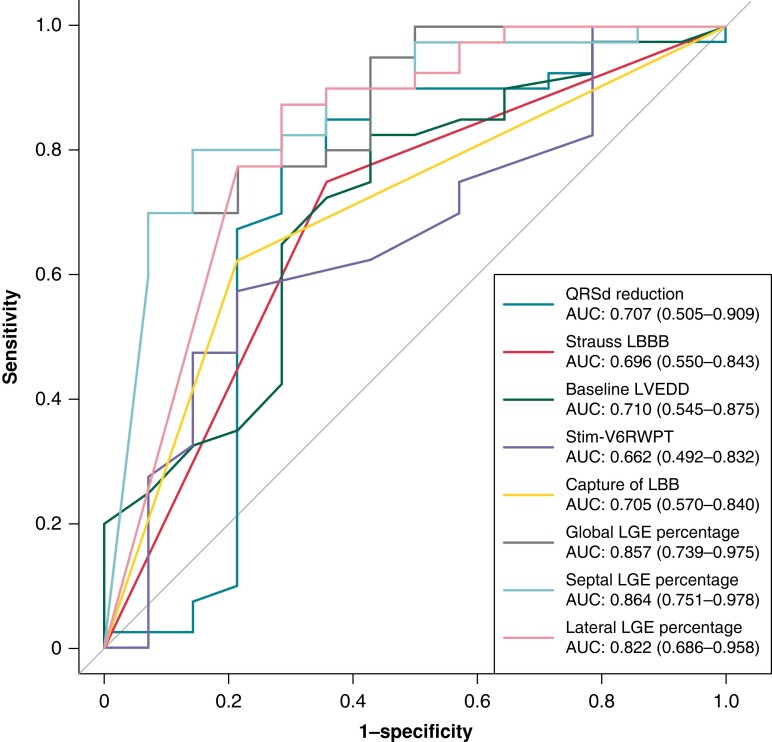
Receiver operating curves of clinical and CMR parameters for predicting echocardiographic response. LBBB, left bundle branch abnormality; LBB, left bundle branch; LVEDD, left ventricular end-diastolic diameter; LGE, late gadolinium enhancement; RWPT, R wave peak time.

**Figure 4:**
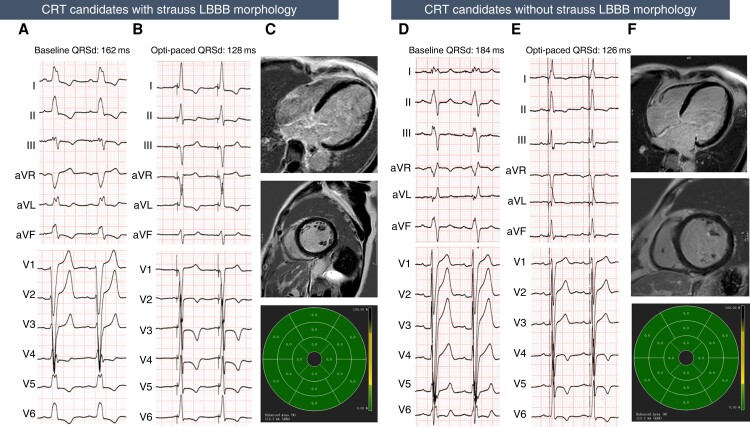
Example of ECG and CMR of LBBAP-CRT responder in patients with or without Strauss LBBB. (*A*–*C*) are from an LBBAP-CRT responder with baseline ECG that shows wide QRS complex (162 ms) and meets the Strauss LBBB criteria (*A*). After LBBAP, optimized paced QRSd was reduced to 128 ms. (*B*) The LVEF recovered from 29% at baseline to 66% and LVESV reduced from 193 to 47 mL after 6 months follow-up. The CMR imaging showed no presence of LGE in the left ventricle (*C*). (*D*–*F*) are from an LBBAP-CRT responder with wide QRS complex (184 ms) but did not meet the Strauss LBBB criteria (*D*). After LBBAP-CRT, QRS complex narrowed to 126 ms (*E*). The LVEF improved from 26% at baseline to 48% and LVESV reduced from 112 to 48 mL after 6 months follow-up. The CMR imaging showed no presence of LGE in the left ventricle (*F*). CRT, cardiac resynchronization therapy; LBBB, left bundle branch abnormality; LVEDD, left ventricular end-diastolic diameter; LVEF, left ventricular ejection fraction; LVESV, left ventricular end-systolic volume; LGE, late gadolinium enhancement; opti-paced QRSd, optimized paced QRS duration.

The predictive values of clinical and imaging variables for echocardiographic response, super-response, and clinical response were evaluated by ROC analyses. The clinical parameters displayed only fair to moderate discrimination for predicting echocardiographic response, with AUCs around 0.7 (*Figure 3* and *Table 4*) and held poor predictive value for super-response (AUC: Strauss LBBB 0.515, baseline LVEDD 0.661, stim-V6 RWPT 0.560, QRSd reduction 0.642, and capture of LBB 0.543). In comparison, the CMR-derived scar percentage displayed very good discriminability for identifying LBBAP-CRT echocardiographic responders, with AUC of 0.864, 0.857, and 0.822 for septal, global, and lateral LGE percentage. Using cut-off values based on the Youden index, global LGE percentage had the specificity of 92.9% and positive LR as high as 9.859, while lateral LGE percentage gives high sensitivity of 87.5% with the negative LR as low as 0.175. And at the 6.99% cut-off value for septal LGE percentage, lower septal scar burden predicted response with a sensitivity of 80% and specificity of 85.7%, with a positive LR of 5.594 and a negative LR of 0.233. Based on the cut-off values noted above, LVEF improvement as well as LVESV and LVEDD reduction were also significantly higher in those with lower global, septal, and lateral scar burden groups (see Supplementary material online, *Figure S2*). When further dividing patients according to Strauss LBBB and global, septal, and lateral LGE percentage (see Supplementary material online, *Figure S3*), we observed that among the patients with the Strauss LBBB morphology, those having lower global, lateral, or septal scars displayed greater LVESV reduction and higher LVEF improvement as compared with the higher septal scar group, while patients who did not meet the Strauss LBBB criteria and having higher global, septal, or lateral scar tend to had less LVEF improvement and LVESV reduction as compared with those having lower scar burdens. *Figure 4* illustrates examples of ECG and CMR images of responders from patients with and without Strauss LBBB morphology. Considering clinical response and super-response, the scar burden also displayed moderate-to-good predictive value, with septal LGE displaying better performance in identifying clinical responders (AUC 0.791) and global LGE demonstrating the highest AUC for predicting super-response (AUC 0.758).

**Table 4:** Clinical and CMR parameters for predicting echocardiographic response

Predictors	AUC (95% CI)	Cut-off	Sensitivity	Specificity	PPV	NPV	Positive LR	Negative LR
Clinical predictors								
QRSd reduction (ms)	0.707 (0.505–0.909)	24.5	0.850	0.643	0.872	0.600	2.381	0.233
Strauss LBBB	0.696 (0.550–0.843)	1	0.750	0.643	0.857	0.474	2.101	0.389
Baseline LVEDD (mm)	0.710 (0.545–0.875)	70.5	0.825	0.571	0.846	0.533	1.923	0.306
Stim-V6 RWPT (ms)	0.662 (0.492–0.832)	91.5	0.575	0.786	0.885	0.393	2.687	0.541
Capture of LBB	0.705 (0.570–0.840)	1	0.625	0.786	0.893	0.423	2.921	0.477
CMR predictors								
Global LGE percentage (%)	0.857 (0.739–0.975)	1.77	0.700	0.929	0.966	0.520	9.859	0.323
Septal LGE percentage (%)	0.864 (0.751–0.978)	6.99	0.800	0.857	0.941	0.600	5.594	0.233
Lateral LGE percentage (%)	0.822 (0.686–0.958)	0.412	0.875	0.714	0.897	0.667	3.059	0.175

AUC, area under the curve; PPV, positive predictive value; NPV, negative predictive value; LR, likelihood ratio; other abbreviations as in *Tables 1* and *2*.

### Predictive value of scar for adverse clinical outcomes

**Figure 5:**
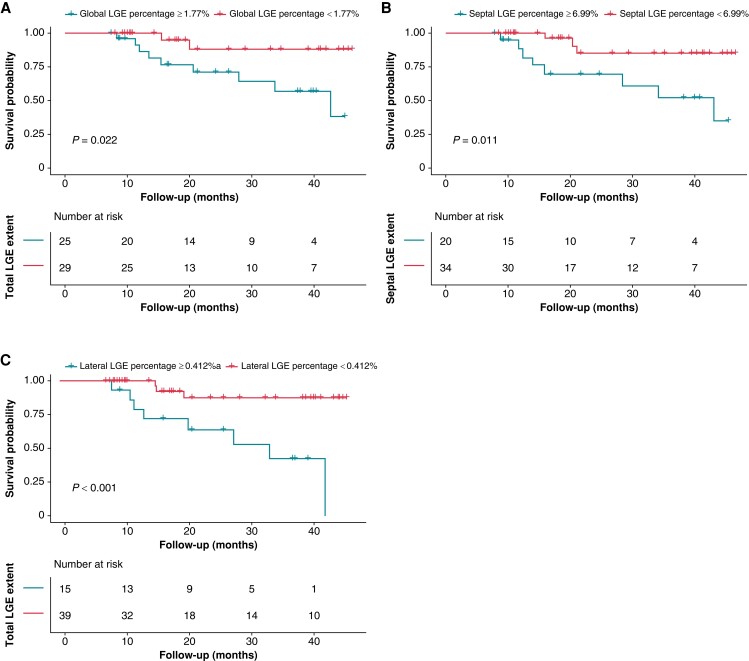
Survival without composite outcomes after LBBAP-CRT in patients with lower and higher myocardial scar. Time to all-cause mortality or heart failure hospitalization according to (*A*) global LGE percentage, (*B*) septal LGE percentage, and (*C*) lateral LGE percentage stratified by cut-off value to predict response in ROC analysis. LGE, late gadolinium enhancement.

Over a median follow-up time of 20.3 (IQR: 11.5–38.7) months, 11 (20.4%) patients reached the combined endpoint of all-cause mortality (*n* = 2) or HFH (*n* = 9). On Kaplan–Meier analysis, there was a significant difference in event-free survival between lower and higher scar burden groups (*Figure 5*). Using the same cut-off value in response analysis for global, septal, and lateral LGE percentage (1.77%, 6.99%, and 0.412%, respectively), the occurrence of the composite outcome differed significantly between patients based on global/septal/lateral scar [HR of higher global scar: 4.996; 95% confidence interval (CI): 1.078–23.151; *P* = 0.040; HR of higher septal scar 4.741; 95% CI: 1.255–17.917, *P* = 0.022; HR of higher lateral scar 7.019; 95% CI: 1.838–26.806, *P* = 0.004; see Supplementary material online, *Table S5*].

## Discussion

This study demonstrated the relationship between CMR-derived scar parameters and 6-month echocardiographic reverse remodelling, as well as clinical prognosis in patients with LBBAP-CRT. There are four important outcomes from this study: First, global, septal, and lateral scar burdens correlated negatively with reverse remodelling and displayed better performance for predicting echocardiographic response than Strauss LBBB morphology (Graphical Abstract). Second, measures of scar burden were also strong predictors of clinical outcomes after LBBAP-CRT. Third, patients with Strauss LBBB morphology tend to have lower septal scar burden compared with those without Strauss LBBB morphology. Fourth, the magnitude of reverse remodelling as measured by changes in LVEF, LVEDD, and LVESV is less in the presence of greater LV scar.

### Traditional clinical parameters

Since Huang *et al.*^26^ reported the first successful case of LBBAP for CRT in an HF patient with LBBB in 2017, variable clinical studies have shown that the feasibility and efficacy of LBBAP are novel pacing approaches and useful for a CRT approach in patients with traditional BVP-CRT indication.^10,27–30^ However, to date, the investigation into predictors of the LBBAP-CRT responders is limited. Although the shorter paced QRSd, greater QRSd reduction, and changes of repolarization parameters were reported to be associated with LBBAP-CRT response,^31,32^ these indices can only be collected during or after the procedure rather than prospectively guide the optimal patient selection. Intuitively, patients with LBBB morphology may benefit from LBBAP, given that the electrical dyssynchrony induced by LBBB can be corrected. Strauss LBBB has been considered as a predictor of ‘true’ LBBB and yielded up to 90% CRT response rate in previous studies,^27,33^ thus being commonly recognized as an important factor in patient election for LBBAP-CRT. However, the utility of the Strauss LBBB criteria may be limited by significant interobserver variability of ECG classification.^34^ Moreover, among patients without LBBB morphology, ∼50–60% response rates were observed previously.^35^ In our study, we observed a higher response rate of in patients with Strauss LBBB than those without (response rates: 85.7% vs. 52.6%, respectively), which re-affirms the usefulness of ECG morphology in identifying a target population who might benefit from LBBAP-CRT. However, the NPV and the specificity of this categorical parameter are only 47.4% and 64.3%, respectively, indicating that this parameter alone is insufficient. Accordingly, patients without typical LBBB morphology may also benefit from LBBAP-CRT more frequently than previously claimed. Although other clinical parameters such as baseline LVEDD and QRS reduction are associated with LVEF reduction in the current study, they are not strong of predicting the clinical response and super-response. These findings suggest that traditional clinical parameters related to electrical synchrony and echocardiographic parameters may not be sufficient to estimate response to LBBAP-CRT accurately.

### Cardiovascular magnetic resonance–derived scars

Beyond the morphological and electrical characteristics, myocardial scar may also impact the effects of CRT.^36,37^ For example, pacing over the scarred myocardial tissue in the posterolateral LV is associated with non-response to traditional BVP-CRT,^13^ which motivated the investigations of the relationship between myocardial fibrosis and LBBAP. A previous retrospective studys with small sample sizes observed that the presence and burden of septal scar is a major factor impeding lead advancement to the left bundle area. High scar burden is strongly associated with failure of LBB pacing.^14^ Regarding the acute haemodynamic response, a small study showed that septal scar attenuated the response to LBBAP in eight patients who underwent pre-procedure CMR.^38^ These studies suggest that scar may affect activation of the left bundle or limit the extent of the conduction system that can activate the myocardium. The present study is the first to analyse the effect of scar features comprehensively on the prognosis after LBBAP-CRT. In this population with relatively lower global (6%) and septal scar burdens (10.7%), who have successful lead implantation in the LBB area, we observed that higher scar percentage was associated with lower likelihood of echocardiographic and clinical response. Furthermore, responders tend to have an absence or lower burden of myocardial scar, whereas non-responders have a higher burden of global, septal, and lateral scars. In multivariate linear regression, the myocardial scar percentage in septum was also strongly correlated with change of LVEF. These findings suggest that the myocardial scars limits the capacity of the LV to remodel independent of improved electrical resynchronization.

In the ROC analysis, scar burden was predictive of response and super-response, with the septal scar displaying the best predictive value for responders (AUC of 0.864), which is higher than other clinical variables. The −LR of all the scar parameters are lower than Strauss LBBB, while the +LR are higher than Strauss LBBB, suggesting that scar parameters have stronger predictive value to classify a responder and non-responder more correctly than ECG morphology.

The assessment of clinical outcomes also indicated that a higher scar burden was associated with an increased risk of death or HFH after LBBAP-CRT. This supports the concept that the uncoupling of LBBB correction and outcome in some patients is due to the poor substrate for mechanical resynchronization. Consequently, myocardial scar is closely related to the adverse remodelling in HF and a marker of the severity of the disease. These findings highlight the prognostic value of CMR-LGE for LBBAP-CRT and suggest that myocardial fibrosis assessment may serve as a sensitive marker with higher discriminability of response and risk prediction in patients who plan to receive LBBAP-CRT. The approach of pre-procedure CMR analysis offers potential values for clinicians to improve the selection of LBBAP-CRT candidates and optimize clinical decisions.

### Limitations

The results of this study should be interpreted in light of certain methodological limitations. First, there was a relatively small sample size and this was a retrospective study. As with all retrospective studies, there may be some selection bias. Secondary, only patients with CMR examination were included in this study, which might also introduce selection bias. In our centre, CMR was routinely recommended for patients with NICM and intended for better understanding of the aetiology of cardiomyopathy. Therefore, the population evaluated was largely a non-ischaemic cohort, so the results of the present study cannot be extrapolated to ischaemic heart disease. Finally, the small number of composite events limits further multivariate adjustment of clinical parameters. Therefore, the independent effect of scar burden on echocardiographic response as well as clinical outcome should be further explored in larger populations with long-term follow-up.

## Conclusions

Lower scar burden is a strong predictor of LBBAP-CRT response. The pre-procedure CMR scar evaluation may provide useful information beyond Strauss LBBB to help clinicians for assessing potential responders to LBBAP-CRT. Future prospective studies are warranted to further explore the efficacy of pre-procedure CMR scar evaluation for optimizing the selection of LBBAP-CRT candidates in both ischaemic and non-ischaemic cohorts.

## Supplementary Material

euad326_Supplementary_Data

## Data Availability

The data will be available upon reasonable request to the corresponding author.
