# Supplementary material for: Cardiac magnetic resonance–derived myocardial scar is associated with echocardiographic response and clinical prognosis of left bundle branch area pacing for cardiac resynchronization therapy
Source: Europace. 2023 Oct 31;25(11):euad326. doi: 10.1093/europace/euad326 (PMC10639094; doi:10.1093/europace/euad326)

Supplementary materials

**Supplementary Method**

**1.****LBB capture**

Unipolar-tip paced QRS morphology was monitored and the pacing stimulus to R wave peak time (stim-RWPT) in lead V6 was measured at both low (≤2V@0.5 ms) and high outputs (≥5V@0.5 ms). LBB capture was considered present according to previous definition as following: If unipolar right bundle branch delay configuration was observed and 1 or more of the following criteria were met: 1) LBB potential with injury current; 2) a transition from non-selective LBB capture to selective LBB capture observed with a reduction of pacing output 3) a transition from non-selective LBB capture to left ventricular (LV) septal capture observed (delta stim-RWPT ≥10ms) with reduction of pacing output; 4) the short and constant stim-RWPT at low and high output (≤ 100ms). LV septal pacing was considered to be present if LBB capture could not be confirmed using the preceding criteria.

**2.** **CMR basic parameters acquisition**

Typical imaging parameters were as follows: field of view (FOV) = 320 × 320mm, matrix = 192 × 224, repetition time (TR)/echo time (TE)=3.3/1.7ms, flip angle=50◦, number of cardiac frames = 25 per cardiac cycle, slice thickness = 8mm, slice gap = 2mm.

Basic cardiac functional and volumetric parameters, including LVEF, cardiac output, left ventricular mass, stroke volume, left ventricular end‐diastolic (or systolic) volume index (LVEDVI, LVESVI) were automatically generated. The late gadolinium enhancement (LGE) images were obtained at the same position as cine images in end-diastole using segmented phase-sensitive inversion recovery (PSIR) sequence 10–15 min after the administration of 0.15 mmol/kg gadolinium-based contrast agent (Magnevist; Bayer Healthcare Pharmaceuticals, Wayne, NJ). Typical imaging parameters included: FOV = 380 × 320mm, matrix = 256 × 162, TR / TE = 8.6/3.36ms, flip angle = 25◦, slice thickness = 8mm, slice gap = 2mm, nominal inversion time = 300-350 ms.

**Table S1 Pacing, echocardiographic and functional measures at implantation and 6 months follow-up**

|  | **All (n=54)** | **Non-Responders (n=14)** | **Responders (n=40)** | ***P* value** |
| --- | --- | --- | --- | --- |
| Evidence of LBB capture, n (%) | 28 (51.9%) | 3 (21.4%) | 25 (62.5%) | 0.008 |
| Stim-V6RWPT, ms | 92.0 (80.0-96.0) | 94.0 (92.0-96.0) | 88.5 (78.2-95.2) | 0.073 |
| Paced QRSd, ms | 135.7 ± 15.9 | 135.2 ±20.4 | 135.9±14.3 | 0.895 |
| QRSd reduction, ms | 33.5 (23.2-44.8) | 14.0 (7.8-29.8) | 37.5 (28.5-45.2) | 0.022 |
| Ventricular pacing percentage, % | 98.1 ± 2.4 | 97.5 ±2.9 | 98.4±2.1 | 0.240 |
| **Echocardiographic parameters** | |  |  |  |
| **LVEF, %** |  |  |  |  |
| Baseline | 30.0 (26.2-35.0) | 28.5 (27.0-32.0) | 30.5 (26.0-35.0) | 0.699 |
| 6 months follow-up | 43.5 (32.8-53.8) * | 27.5 (24.0-32.0) | 47.5 (42.0-55.2)* | <0.001 |
| **LVEDD, mm** |  |  |  |  |
| Baseline | 66.5 (62.0-71.8) | 71.5 (65.0-78.0) | 65.0 (61.8-69.2) | 0.020 |
| 6 months follow-up | 58.0 (52.3-65.0) * | 67.5 (64.2-74.2) | 56.0 (50.0-60.0)* | <0.001 |
| **LVESV, ml** |  |  |  |  |
| Baseline | 163.5 (125.2-193.8) | 195.5 (157.2-263.8) | 154.0 (124.2-184.0) | 0.030 |
| 6 months follow-up | 109.0 (58.0-154.0) * | 180.0 (154.0-273.8) | 91.0 (52.5-120.2)* | <0.001 |
| **Mitral regurgitation (0-4)** |  |  |  |  |
| Baseline | 2.2 ± 1.1 | 2.8±1.1 | 2.0±1.0 | 0.031 |
| 6 months follow-up | 1.4 ± 1.1 * | 2.4±1.1 | 1.1±0.9* | <0.001 |
| **Tricuspid regurgitation (0-4)** |  |  |  |  |
| Baseline | 1.3 ± 0.9 | 1.7±0.8 | 1.2±0.9 | 0.069 |
| 6 months follow-up | 1.2 ± 0.9 | 1.6±0.9 | 1.0±0.9 | 0.051 |
| **NYHA functional class** |  |  |  |  |
| Baseline | 2.8 ± 0.5 | 3.1±0.55 | 2.7±0.5 | 0.013 |
| 6 months follow-up | 1.8 ± 0.8 | 2.8±0.7 | 1.5±0.6* | <0.001 |
| **Clinical responders**, n (%) | 35(64.8%) | 1 (7.1%) | 34 (85.0%) | <0.001 |
| **Super responders**, n (%) | 21(38.9%) | 0 (0.0%) | 21 (52.5%) | <0.001 |

LBB= left bundle branch, Stim-V6RWPT= pacing stimulus to R wave peak time in lead V6, LVEDD= left ventricular end diastolic diameter, LVEF= left ventricular ejection fraction, NYHA= New York heart association, * indicates significant difference (p<0.05) compare with baseline parameter.

**Table S2. Comparison of CMR imaging and Scar features of left ventricle between echocardiographic responders and non-responders.**

|  | **ALL patients** | **Non-Responders (n=14)** | **Responders (n=40)** | **P-value** |
| --- | --- | --- | --- | --- |
| **CMR basic parameters** | |  |  |  |
| LVEF, % | 27.5 (23.2-34.0) | 26.0 (23.0-32.8) | 28.0 (25.5-34.2) | 0.264 |
| Cardiac output, L/min | 5.1 (3.6-6.2) | 4.6 (3.4-6.2) | 5.2 (3.9-6.1) | 0.547 |
| Left ventricle mass, g | 128.1 (104.9-167.7) | 119.2 (106.9-140.0) | 131.2 (105.0-168.7) | 0.693 |
| LVEDVI, ml/m2 | 172.1 (138.3-213.1) | 165.5 (118.1-271.8) | 172.1 (152.1-210.2) | 0.969 |
| Stroke Volume, ml | 83.7 (68.2-97.6) | 80.4 (67.5-98.2) | 88.9 (68.4-97.5) | 0.693 |
| **Scar presence** |  |  |  |  |
| Absent, n (%) | 25(46.3%) | 1 (7.1%) | 24 (60.0%) | <0.001 |
| Present, n (%) | 29(54.7%) | 13 (92.9%) | 16 (40.0%) |  |
| **Scar percentage, %** |  |  |  |  |
| Global, % | 1.0 (0.0-6.4) | 12.2 (4.3-25.4) | 0.0 (0.0-3.1) | <0.001 |
| Lateral, % | 1.3 (0.0-11.4) | 5.2 (0.2-38.2) | 0.0 (0.0-0.0) | <0.001 |
| Septal, % | 0.0 (0.0-0.9) | 14.5 (8.5-21.9) | 0.0 (0.0-4.5) | <0.001 |
| **Location #** | |  |  |  |
| Free wall/septal only, n (%) | 17(58.6%) | 4 (30.8%) | 13 (81.2%) | 0.006 |
| Both, n (%) | 12(41.4%) | 9 (69.2%) | 3 (18.8%) |  |
| **Scar pattern#** |  |  |  |  |
| Mid-wall, n (%) | 23 (79.3%) | 11 (84.6%) | 12 (75.0%) | 0.663 |
| Subendocardial, n (%) | 9 (31.0%) | 5 (38.5%) | 4 (25.0%) | 0.688 |
| Subepicardial, n (%) | 1 (3.4%) | 1 (7.7%) | 0 (0.0%) | 0.448 |
| Transmural, n (%) | 3 (10.3%) | 2 (15.4%) | 1 (6.2%) | 0.573 |
| **Scar percentage, % #** |  |  |  |  |
| Global, % | 6.0 (3.0-13.6) | 16.6 (4.3-27.0) | 5.1 (2.0-7.0) | 0.022 |
| Lateral, % | 0.7 (0.0-11.6) | 8.6 (0.7-39.7) | 0.1 (0.0-1.8) | 0.017 |
| Septal, % | 10.7 (4.6-17.3) | 17.3 (8.8-22.2) | 7.4 (3.8-11.8) | 0.013 |
| Basal septal, % | 11.4 (2.1-16.9) | 14.8 (10.9-38.9) | 6.2 (1.3-14.0) | 0.028 |
| Mid septal, % | 8.9 (5.8-15.3) | 11.7 (8.7-17.8) | 7.7 (3.1-11.3) | 0.059 |
| Apical septal, % | 0.0 (0.0-1.8) | 0.0 (0.0-10.9) | 0.0 (0.0-1.3) | 0.408 |

# values were only summarized from 29 patients who have the myocardial LGE present.

LVEF= left ventricular ejection fraction, LVED(S)VI= left ventricular end‐diastolic (or systolic) volume index

**Table S3. Clinical and CMR parameters for predicting clinical response**

| Predictors | AUC 95% CI | Cut-off | sensitivity | specificity | PPV | NPV |
| --- | --- | --- | --- | --- | --- | --- |
| **Clinical predictors** |  |  |  |  |  |  |
| QRSd reduction, (ms) | 0.617(0.441-0.793) | 18 | 0.886 | 0.421 | 0.738` | 0.667 |
| Strauss LBBB | 0.635(0.498-0.771) | 1 | 0.743 | 0.526 | 0.743 | 0.526 |
| Baseline LVEDD, (mm) | 0.702(0.553-0.850) | 68.5 | 0.743 | 0.579 | 0.765 | 0.55 |
| Stim-V6 RWPT, (ms) | 0.736(0.604-0.868) | 91.5 | 0.657 | 0.842 | 0.885 | 0.571 |
| Capture of LBB | 0.697(0.568-0.826) | 1 | 0.671 | 0.737 | 0.821 | 0.539 |
| **CMR predictors** |  |  |  |  |  |  |
| Global LGE percentage (%) | 0.779(0.649-0.909) | 1.77 | 0.743 | 0.842 | 0.897 | 0.64 |
| Septal LGE percentage (%) | 0.791(0.662-0.920) | 4.54 | 0.8 | 0.79 | 0.875 | 0.682 |
| Lateral LGE percentage (%) | 0.723(0.585-0.860) | 0.42 | 0.886 | 0.579 | 0.795 | 0.733 |

AUC= area under the curve, PPV= positive predictive value, NPV=negative predictive value, other abbreviations as in Table S1 and S2.

**Table S4. Clinical and CMR parameters for predicting super-response**

| Predictors | AUC 95% CI | Cut-off | Sensitivity | Specificity | PPV | NPV |
| --- | --- | --- | --- | --- | --- | --- |
| **Clinical predictors** |  |  |  |  |  |  |
| QRSd reduction, (ms) | 0.642(0.488-0.798) | 35.5 | 0.619 | 0.667 | 0.542 | 0.733 |
| Strauss LBBB | 0.515(0.356-0.674) | 1 | 0.667 | 0.364 | 0.4 | 0.632 |
| Baseline LVEDD, (mm) | 0.661(0.514-0.808) | 69.5 | 0.905 | 0.485 | 0.528 | 0.889 |
| Stim-V6RWPT, (ms) | 0.560(0.395-0.725) | 79.5 | 0.33 | 0.859 | 0.583 | 0.667 |
| Capture of LBB | 0.543(0.385-0.702) | 1 | 0.571 | 0.515 | 0.515 | 0.654 |
| **CMR predictors** |  |  |  |  |  |  |
| Global LGE percentage (%) | 0.758(0.641-0.875) | 3.15 | 0.905 | 0.576 | 0.576 | 0.905 |
| Septal LGE percentage (%) | 0.742(0.621-0.863) | 0.002 | 0.714 | 0.697 | 0.6 | 0.793 |
| Lateral LGE percentage (%) | 0.735(0.631-0.838) | 0.001 | 0.905 | 0.546 | 0.559 | 0.900 |

AUC= area under the curve, PPV= positive predictive value, NPV=negative predictive value, other abbreviations as in Table S1 and S2.

**Table S5. Univariate cox analysis**

| **Parameters** | **HR** | **95%CI lower** | **95%CI upper** | **P value** |
| --- | --- | --- | --- | --- |
| LGE percentage<1.77% | Reference |  |  |  |
| LGE percentage≥1.77% | 4.996 | 1.078 | 23.151 | 0.040 |
| Septal LGE percentage <6.99% | Reference |  |  |  |
| Septal LGE percentage≥6.99% | 4.741 | 1.255 | 17.917 | 0.022 |
| Lateral LGE percentage<0.412% | Reference |  |  |  |
| Lateral LGE percentage≥0.412% | 7.019 | 1.838 | 26.806 | 0.004 |

HR= hazard ratio, other abbreviations as in Table S1 and S2

**Figure S1.** Flow-chart of patient enrollment

**
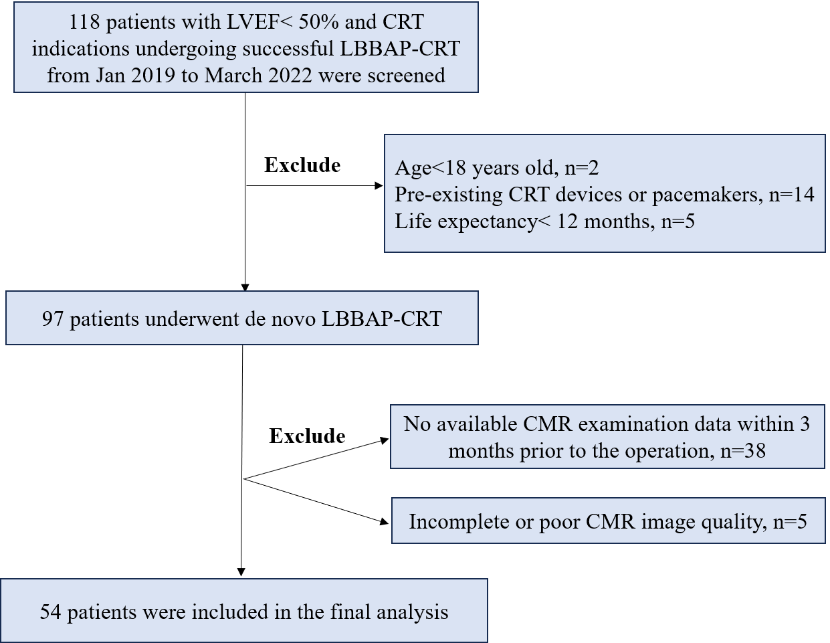
**

**Figure S2. Comparison of LVEF, LVESV and LVEDD changes according to scar burden.**

Comparison of LVEF improvement (A-C), percentage of LVESV reduction (D-F) and LVEDD reduction (G-I) between patients with low and high global septal, lateral LGE percentage based on the cut-off value derived from ROC analysis for echocardiographic response (1.77% for global LGE, 6.99% for septal LGE and 0.412% for septal LGE based on Youden index of ROC analysis to divide the low and high global, septal and lateral scar).

**
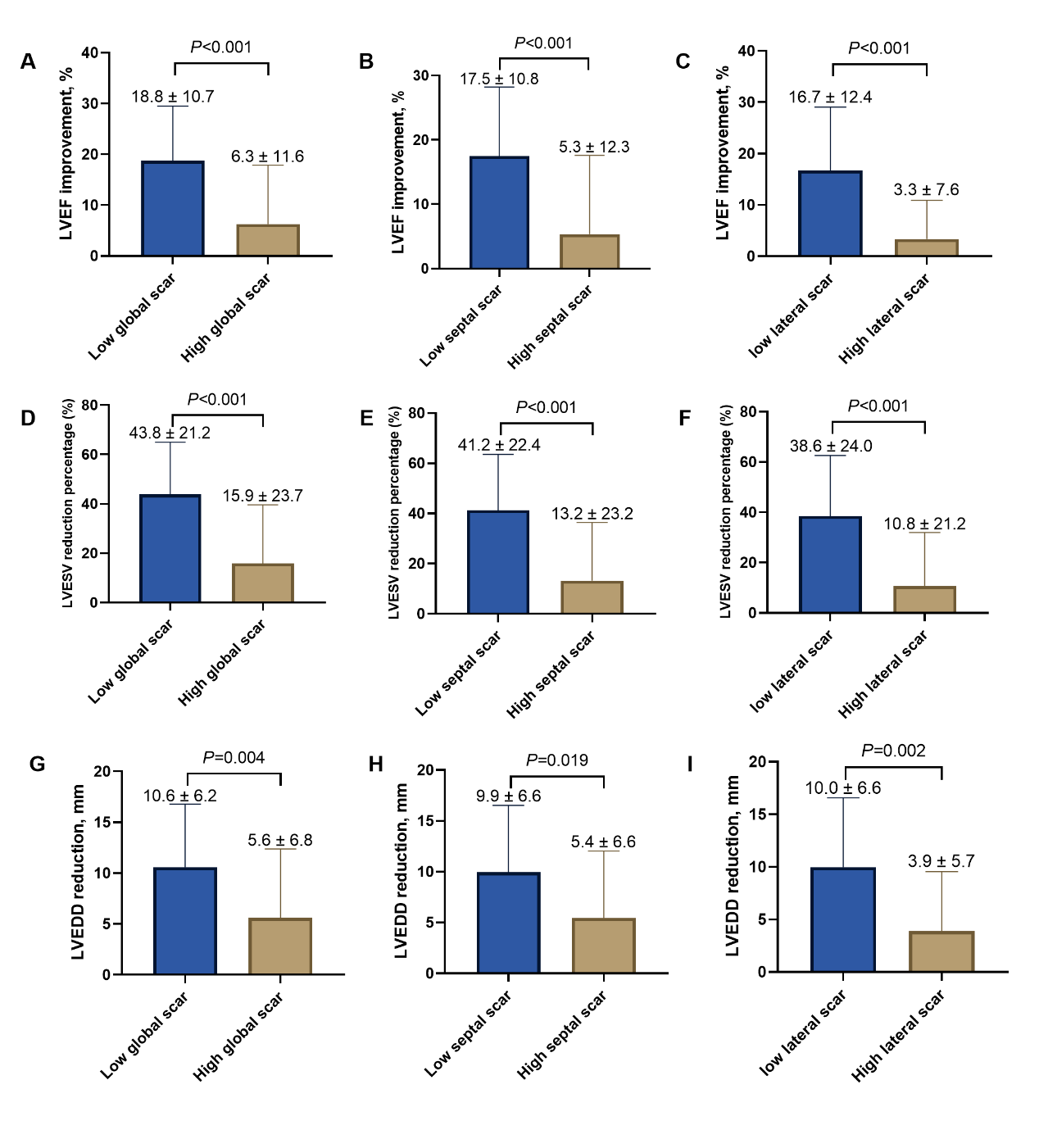
**

**Figure S3. LVEF and LVESV change at 6 months according to septal scar burden and Strauss LBBB morphology**

Comparison of LVEF improvement (A-C), percentage of LVESV reduction (D-F) between different groups divided by global, lateral and septal LGE percentage and Strauss LBBB morphology. (1.77% for global LGE, 6.99% for septal LGE and 0.412% for septal LGE based on Youden index of ROC analysis to divide the low and high global, septal and lateral scar)


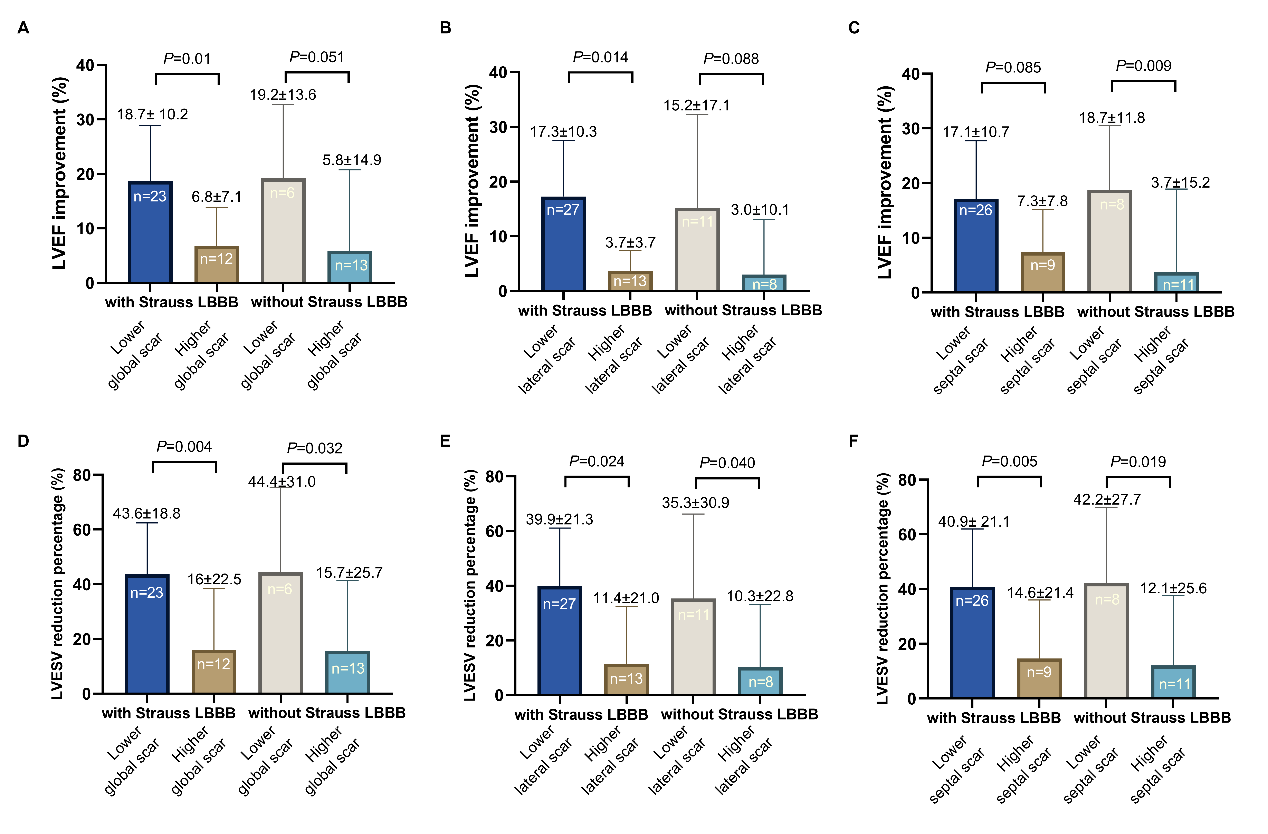

Supplement: euad326_Supplementary_Data [file euad326_supplementary_data.docx]
